# Assessing the Role of Developmental and Environmental Factors in Chemical Defence Variation in Heliconiini Butterflies

**DOI:** 10.1007/s10886-021-01278-7

**Published:** 2021-05-18

**Authors:** Ombeline Sculfort, Melanie McClure, Bastien Nay, Marianne Elias, Violaine Llaurens

**Affiliations:** 1 Institut de Systématique, Evolution, Biodiversité (ISYEB), Muséum National D’Histoire Naturelle, CNRS, Sorbonne-Université, EPHE, Université Des Antilles, 45 rue Buffon, 75005 Paris, France; 2 Unité Molécules de Communication Et Adaptations Des Micro-Organismes (MCAM), Muséum National D’Histoire Naturelle, CNRS, 57 rue Cuvier (BP 54), 75005 Paris, France; 3 Laboratoire Écologie, Évolution, Interactions Des Systèmes Amazoniens (LEEISA), Université de Guyane, CNRS, IFREMER, 97300 Cayenne, France; 4 Laboratoire de Synthèse Organique, Ecole Polytechnique, CNRS, ENSTA, Route de Saclay, 91128 Palaiseau Cedex, France

**Keywords:** Biosynthesis, Cyanogenic glucosides, Heliconiini, Sequestration

## Abstract

Chemical defences in animals are both incredibly widespread and highly diverse. Yet despite the important role they play in mediating interactions between predators and prey, extensive differences in the amounts and types of chemical compounds can exist between individuals, even within species and populations. Here we investigate the potential role of environment and development on the chemical defences of warningly coloured butterfly species from the tribe Heliconiini, which can both synthesize and sequester cyanogenic glycosides (CGs). We reared 5 Heliconiini species in captivity, each on a single species-specific host plant as larvae, and compared them to individuals collected in the wild to ascertain whether the variation in CG content observed in the field might be the result of differences in host plant availability. Three of these species were reared as larvae on the same host plant, *Passiflora riparia*, to further test how species, sex, and age affected the type and amount of different defensive CGs, and how they affected the ratio of synthesized to sequestered compounds. Then, focusing on the generalist species *Heliconius numata*, we specifically explored variation in chemical profiles as a result of the host plant consumed by caterpillars and their brood line, using rearing experiments carried out on two naturally co-occurring host plants with differing CG profiles. Our results show significant differences in both the amount of synthesized and sequestered compounds between butterflies reared in captivity and those collected in the field. We also found a significant effect of species and an effect of sex in some, but not all, species. We show that chemical defences in *H. numata* continue to increase throughout their life, likely because of continued biosynthesis, and we suggest that variation in the amount of synthesized CGs in this species does not appear to stem from larval host plants, although this warrants further study. Interestingly, we detected a significant effect of brood lines, consistent with heritability influencing CG concentrations in *H. numata*. Altogether, our results point to multiple factors resulting in chemical defence variation in Heliconiini butterflies and highlight the overlooked effect of synthesis capabilities, which may be genetically determined to some extent.

## Introduction

Predation is an important selective force and a wide range of defensive traits have evolved in different prey species, including chemical defences (Ruxton et al. 2004). Among insects, defensive compounds can be synthesized de novo (e.g. ants Touchard et al. 2015) or sequestered from the diet (e.g. monarch butterflies Brower et al. 1967), although the two processes are not mutually exclusive. In organisms that sequester chemical defences from their diet, differences in the chemical composition or availability of these resources can result in variable defences (Jones et al. 2019). In prey that biosynthesize defensive compounds, the amount of defences can be constrained as a result of trade-offs in the allocation of limiting resources (Zvereva et al. 2017). Genetic or other inheritable differences (e.g. maternal effect) can also explain variation in defences in both organisms that biosynthesize (Alape-Girón et al. 2008) and those that sequester compounds (Fordyce and Nice 2008). Finally, regardless of how defensive compounds are obtained, chemical defences can also vary according to sex (Jeckel et al. 2015a, b; Kissner et al. 2000; Nahrstedt and Davis 1983), developmental stage (Columbus-Shenkar et al., 2018; Dossey et al., 2008), and age (Jeckel et al. 2015a, b; Hayes et al. 2009; Zagrobelny et al. 2007a, b). Such variation can stem from physiological and/or ecological differences, such as differences in diet, and as a result, large qualitative and quantitative variation in chemical defences can occur between individuals, even within a given population or species (Alape-Girón et al. 2008; María Arenas et al. 2015; McGugan et al. 2016; Sculfort et al. 2020).

**Fig. 1:**
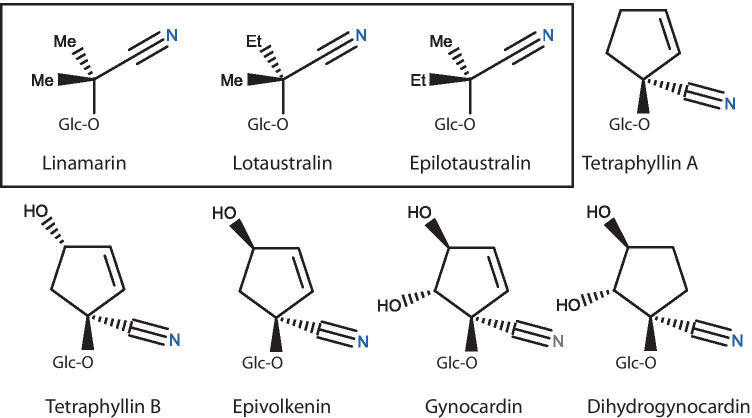
Reported cyanogenic glucosides within Heliconiini butterflies. The three aliphatic CGs putatively-synthesized by Heliconiini are shown in the box above, followed by five cyclopentenoid CGs sequestered from *Passiflora* plants. Abbreviations: *Et* ethyl group (CH_2_CH_3_), *Me* methyl group (CH_3_), *Glc* glucose.

Extensive variation in chemical compounds has been observed in butterflies of the neotropical tribe Heliconiini, whereby different species, but also different geographic populations within species, differ in their chemical defences (Hay-Roe 2004; Sculfort et al. 2020). These brightly coloured aposematic butterflies are chemically defended by cyanogenic glycosides (hereafter CGs). These butterflies release toxic cyanide, shown to be a deterrent to predators, through a process called cyanogenesis (Cardoso 2020; Poulton 1990). These compounds can be sequestered from the butterflies’ *Passiflora* host plants as larvae (Engler et al. 2000), synthesized de novo (Wray et al. 1983), or both (de Castro et al. 2020; Hay-Roe 2004). Heliconiini are indeed known to de novo synthesize two aliphatic CGs (a CG with two simple linear radical chains), namely linamarin and lotaustralin, as both larvae and adults (Fig. 1) (Davis and Nahrstedt, 1987; Nahrstedt and Davis 1985; Wray et al. 1983). A third aliphatic CG, epilotaustralin, was reported in wild Heliconiini and has been hypothesized to be de novo synthesized concomitantly with its lotaustralin epimer (Sculfort et al. 2020). However, at least five *Passiflora* species consumed by some Heliconiini also produce these three aliphatic CGs (de Castro et al. 2019; Jiggins 2016; Olafsdottir et al. 1989) and captive-reared caterpillars of *Heliconius melpomene*, for example, have been shown to sequester these aliphatic CGs (de Castro et al. 2019). As we are currently unable to determine the metabolic origin of these compounds in adult butterflies, we will use the term “putatively-synthesized” hereafter when referring to these CGs. Approximately 30 other kinds of CGs have also been reported in *Passiflora* species (Bombardelli et al. 1975; Clausen et al. 2002; de Castro et al. 2017), and Heliconiini butterflies have been shown to sequester several of them (de Castro et al. 2019; Engler et al. 2000; Hay-Roe 2004; Sculfort et al. 2020) (Fig. 1).

How butterflies obtain CGs may be linked to host plant specialization, with sequestration found to be more predominant in specialist species (Engler and Gilbert 2007). For example, specialist species were found to have higher total concentrations of CGs when consuming their preferred host plant as larvae, whereas generalists had lower overall total concentrations of CGs that depended less on the host plant ( Arias et al. 2016; de Castro et al. 2019; Engler and Gilbert 2007; Sculfort et al. 2020 but see Hay-Roe and Nation 2007). Differences in host plant use and/or diet breadth as larvae could therefore explain some of the chemical differences observed both between species and within wild populations of Heliconiini (Arias et al. 2016; Sculfort et al. 2020). However, these differences could also stem from differences in physiology, metabolism, or other inheritable differences (e.g. genetic and/or maternal effect). Furthermore, rearing experiments with *H. melpomene* have shown continued de novo synthesis throughout the butterflies’ lives, resulting in a net increase in CG quantities with age ( de Castro et al. 2020; Nahrstedt and Davis 1985). Individual differences in wild populations may therefore be the result of aging and continued accumulation of these compounds as well as amounts sequestered during the larval stage. To test which factors are responsible for the variation in chemical defences observed within and between natural populations, and between species, controlled rearing experiments using different larval host plants and Heliconiini species are needed.

Here we compared the chemical defences of five Heliconiini species, each reared on a single species-specific host plant as larvae, and those of wild-caught individuals, to ascertain whether the chemical variation was still observed in captivity and whether this was the result of synthesized and/or sequestered compounds. We also tested how species, sex, and age affected the type and amount of different defensive CGs, and how these affected the ratio of synthesized to sequestered compounds, by rearing 3 Heliconiini species as larvae on the same *Passiflora* host plant species. We further explored variation in chemical profiles (i.e. the assemblage of all CG compounds within an individual) in the generalist Heliconiini species, *H. numata,* reared on two naturally co-occurring host plant species with contrasting CG content. The first larval host plant, *Passiflora edulis*, contains a diversity of CGs from which only epivolkenin and tetraphyllin B (Patel 2009) are known to be sequestered by Heliconiini, whereas the other, *Passiflora riparia*, has no reported CGs (de Castro et al. 2019). This experimental set-up enables the investigation of how larval host plant and/or inherited differences (genetic and/or maternal effect) may explain the variation in chemical defences observed within natural populations.

## Methods and Materials

### Butterfly Samples.

The experiment took place during the dry season, between April and August 2017, in the city of Tarapoto (6° 28′ 50.5" S 76° 21′ 22.4" W; installations built and maintained by one of the authors, M.M, on her private property) in Northern Peru (department of San Martín). Wild butterflies were caught in forested sites near the city of Tarapoto. Most wild-caught individuals were preserved as indicated below, but some females were kept in individual outdoor cages (4.0 × 3.0 × 2.5 m-width × length × height), where they were fed ad libitum with fresh commercial pollen and sugar water and were given access to *Passiflora* host plants for egg-laying. We reared the solitary species *Heliconius erato*, *H. melpomene,* and *H. numata*, and gregarious species *Dione juno* and *H. doris* (Table 1). Eggs were collected daily and larvae of solitary species were kept individually in plastic containers to avoid cannibalism. As caterpillars got larger, they were kept in larger and higher plastic containers, large enough for prepupae to safely pupate and emerge as adults. Gregarious caterpillars of *D. juno* and *H. doris* were reared communally in two small outdoor cages (45 × 45 × 45 cm-width × length × height), and after emergence, adults were separated by sex to avoid mating and transferred to larger outdoor cages (4.0 × 3.0 × 2.5 m-width × length × height). Host plant species used to rear the caterpillars were naturally occurring host plant species used in the field by the respective butterfly species and were sufficiently abundant on site to provide fresh food for the entire caterpillar development. Captive-reared individuals were fed with the selected *Passiflora* species as caterpillars and adult butterflies were fed with fresh pollen, provided as pollen masses collected from honeybees and purchased at a local store, and sugar-water. Age was counted in days from the day of emergence for all captive-reared adults, and all were kept virgin.

**Table 1:** Cg concentrations (mean ± se) of captive-reared adults used in the study

Species Developmental stage	Larval host plant 1		Larval host plant 2	
	Number of individuals	Mean [CG]total	Number of individuals	Mean [CG]total
*Dione juno*	*Passiflora riparia*		**-**	
Adult – female	10	72.82 ± 7.03	-	
Adult – male	10	10.19 ± 2.10		
Adult – total	20	41.50 ± 7.96		
*Heliconius doris*	*Passiflora riparia*		**-**	
Adult – female	13	47.85 ± 4.35	-	
Adult – male	9	37.45 ± 4.86		
Adult – total	22	43.60 ± 3.35		
*Heliconius erato*	*Passiflora trifasciata*		-	
Adult – male	5	8.34 ± 5.61	-	
*Heliconius erato*	*Passiflora trifasciata*		-	
Adult – female	3	4.70 ± 1.92	-	
Adult – male	1	0		
Adult – total	4	2.96 ± 1.80		
*Heliconius melpomene*	*Passiflora triloba*		-	
Adult – female	3	33.44 ± 8.20	-	
Adult – male	2	14.06 ± 5.11		
Adult – total	5	25.69 ± 7.61		
*Heliconius numata*	*Passiflora riparia*		-	
Adult – female	12	26.53 ± 4.58	-	
Adult – male	12	33.25 ± 4.59		
Adult – total	24	29.89 ± 3.24		
*Heliconius numata*	*Passiflora riparia*		*Passiflora edulis*	
Adult – female	5	31.10 ± 8.99	3	43.77 ± 17.90
Adult – male	5	39.64 ± 7.81	3	58.61 ± 10.97
Adult – total	10	35.37 ± 13.65	6	51.19 ± 26.13
*Heliconius numata*	*Passiflora riparia*		*Passiflora edulis*	
Adult – female	8	39.16 ± 9.38	11	53.82 ± 8.59
Adult – male	12	41.88 ± 7.76	8	25.53 ± 4.28
Adult – total	20	40.79 ± 6.19	19	41.91 ± 6.94
*Heliconius numata*	*Passiflora riparia*		*Passiflora edulis*	
Adult – female	3	27.30 ± 12.34	9	36.19 ± 6.55
Adult – male	4	13.25 ± 8.96	7	24.18 ± 8.22
Adult – total	7	19.87 ± 6.48	16	30.93 ± 5.48
*Heliconius numata*	*Passiflora riparia*		*Passiflora edulis*	
Adult – female	2	12.71 ± 7.86	3	21.16 ± 4.84
Adult – male	2	66.95 ± 9.39	3	28.00 ± 12.14
Adult – total	4	39.83 ± 14.56	6	24.58 ± 6.42

Five species of Heliconiini were reared on four different species of *Passiflora*. Three of these Heliconiini species were reared on a single *Passiflora* species, *P. riparia*: *D. juno* (1 lineage, *n* 20 adults) and *H. doris* (1 lineage, *n* 22 adults) and *H. numata* (*n* 24 adults). *H. erato* (2 lineages, *n* 4 + 5 adults) was reared on *P. trifasciata* and *H. melpomene* (1 lineage, *n* 5 adults) on *P. triloba*. For four lineages of *H. numata* (*n* 88 adults)*,* broods were separated into two batches to be reared on either *P. riparia* or *P. edulis*

### Cyanogenic Glucoside Extraction in Methanol.

 To extract CGs from adult butterflies, wings were first removed and preserved dry in envelopes (as described in Sculfort et al. 2020). The bodies of butterflies were preserved in individual vials of 100% methanol and kept in the freezer (approximately –18 °C). Extractions were performed at the French National Museum of Natural History in Paris. Methanol was evaporated at room temperature until fully dry using a Savant Automatic Environmental SpeedVac System AES1010 with VaporNet. Air dry butterflies (body + wings) were weighed before being crushed into a fine powder with a mortar and pestle using liquid nitrogen. Two mL of 100% methanol was added to the butterfly powder before stirring for 1 h at room temperature. Extracts were centrifuged for 20 min at 1,600 rotations per minute (4,500 g), filtered using 7 mm diameter glass pipettes and cotton, filtered again with a MultiScreen 0.45 µm hydrophilic low protein binding plate, and centrifuged for 5 min at 3,500 rotations per minute (10,000 g). Raw filtrates were diluted 50 times in deionized water, vortexed, and stored overnight in the refrigerator at 4 °C until used for liquid chromatography and tandem mass spectrometry (LC–MS/MS).

### Liquid Chromatography and Tandem Mass Spectrometry.

 The protocol used has previously been optimized to identify and quantify CGs in butterfly methanol filtrates (Briolat et al. 2019), although a similar method was first applied to *Heliconius* by Hay-Roe (2004). Analytical LC–MS/MS was performed using an Agilent 1100 Series LC (Agilent Technologies, Germany) coupled to a Bruker HCT-Ultra ion trap mass spectrometer (Bruker Daltonics, Germany). Chromatographic separation was carried out on a Zorbax SB-C18 column (Agilent; 1.8 μM, 2.1 × 50 mm). Mobile phase A was composed of deionized water containing 0.1% (v/v) formic acid. Mobile phase B was acetonitrile supplemented with 50 μM NaCl and 0.1% (v/v) formic acid. The gradient was as follows: 0—0.5 min, isocratic 2% B; 0.5—7.5 min, linear gradient 2%—40% B; 7.5—8.5 min, linear gradient 40%—90% B; 8.5—11.5 isocratic 90% B; 11.6—17 min, isocratic 2% B. Flow rate was set up at 0.2 mL/min and increased at 0.3 mL/min between 11.2 to 13.5 min. During the LC step, initially neutral CGs get associated with Na + cation and these molecular ions were analysed with MS in positive electrospray mode. The oven temperature was kept at 35 °C.

In addition to the 284 butterfly samples, we ran blank control samples and reference samples. Blank samples consisted of methanol that had been through the extraction protocol, and the reference sample was a mix of filtrates from every butterfly. Cyanogenic glycosides were identified by comparison to standard solutions (aliphatic CGs were chemically synthesized at the Department of Plant and Environmental Sciences, at the University of Copenhagen, Møller et al. 2016, cyclopentenoid CGs were synthesized and donated by Gilbert and Engler et al. 2000). We made three calibration curves based on three commercial standards: linamarin, lotaustralin/epilotaustralin, and amygdalin (commercial, Sigma Aldrich), from 0.1 to 20 ng/µL each. Blanks, standards, calibration curves, and reference samples were run first. The reference sample was injected after every ten butterfly samples.

### Chemical Data Analyses. 

Mass spectra were analysed using the Bruker Compass Data Analysis 4.3 software. We targeted sodium adducts [M + Na^+^] of linamarin [retention time (RT) 2.4 min at *m/z* = 270], lotaustralin [RT 5.4 min at *m/z* = 284], epilotaustralin [RT 5.5 min at *m/z* = 284], tetraphyllin B [RT 1.3 min at *m/z* = 310], epivolkenin [RT 2.3 min at *m/z* = 310], tetraphyllin A [RT 4.9 min at *m/z* = 294], gynocardin [RT 1.4 min at *m/z* = 326], dihydrogynocardin [RT 1.4 min at *m/z* = 328] and amygdalin [RT 6.4 min at *m/z* = 480]. For every targeted compound, the total amount was estimated based on the Extracted Ion Chromatogram (EIC) peak areas and quantification based on regression equations calculated from the standard curves (in ng of CG/mL of butterfly extract). The concentration of each CG in every butterfly sample was determined by dividing the total amount of compounds by the butterfly dry mass, and the final concentration is given in µg of CG per mg of dry butterfly weight.

### Experimental Design.

 We performed three experiments as described.

### Experiment 1: Differences in Synthesized and Sequestered Compounds in Captive-Reared Versus Wild-Caught Butterfly Species.

 To test whether chemical defence profiles differ between captive-reared butterflies reared on selected host plants as larvae and wild-caught butterflies accessing a potentially larger range of naturally occurring host plants, we reared 5 different Heliconiini species on their preferred local host plant species as larvae (Table 1). This was performed using adults of single broods of *D. juno* (*n* = 20 adults), *H. doris* (*n* = 22), and *H. numata* (*n* = 24) reared on *P. riparia*. A brood of *H. melpomene* (*n* = 5) and two broods (brood_1_ *n* = 4; brood_2_ *n* = 5) of *H. erato* were reared on *P. triloba* and *P. trifasciata* respectively (Table 1). The chemical composition of these individuals was compared to that of wild-caught individuals collected from the same site in Peru (*D. juno n* = 13 adults, *H. doris n* = 8, *H. erato n* = 36, *H. melpomene n* = 25, *H. numata n* = 34). The age of wild-caught butterflies, as well as the host plant species consumed by these larvae, are unknown. However, data on wild-caught butterflies reflects the naturally occurring variance observed in the wild and comparing them to individuals reared in captivity under specific conditions is useful to ascertain how much of the variance observed may be the result of the factors investigated. Nevertheless, because we did not directly assess the CG content of the plants used to feed our captive-reared caterpillars, we cannot rule out a potential chemical variation within *Passiflora* species.

### Experiment 2: Species, Sex, and Age-Related Differences in Chemical Defences when Controlling for Larval Host Plant Use.

 We tested how different intrinsic factors, namely butterfly species, sex, and age, were related to qualitative and quantitative variation in CG content, when controlling for host plant use as larvae. We thus focused on the subsample of virgin adults (*n* = 66 adults in total) of *D. juno* (*n* = 20 adults), *H. doris* (*n* = 22), and *H. numata* (*n* = 24), all raised on the same larval host plant, *P. riparia* (Table 1).

### Experiment 3: Intraspecific Variation in Chemical Defences in Heliconius numata When Fed Two Different Diets.

 The effect of brood line, sex, age, larval diet, and their interactions on intraspecific variation in chemical defences were tested using a total of 88 captive-reared adults from four broods of the generalist species *H. numata*. Eggs of each female were collected and separated into two batches for caterpillars to be reared on either the CG-free *Passiflora riparia* (de Castro et al. 2019) or the CG containing *P. edulis* (Patel 2009), both naturally occurring and widely accepted larval host plants (line_1_ *n* = 10 adults on *P. riparia* and *n* = 6 on *P. edulis*; line_2_ *n* = 20 on *P. riparia* and *n* = 19 on *P. edulis*; line_3_ *n* = 7 on *P. riparia* and *n* = 16 on *P. edulis*; line_4_ *n* = 4 on *P. riparia* and *n* = 6 on *P. edulis*, see Table 1). We sampled virgin captive-reared adults at various times to also test for the effect of age.

### Statistical Analyses.

 All statistics were computed using R 3.4.4 and RStudio 1.1.463 (RStudio Team, 2016; R Core Team, 2017).

### Experiment 1: Differences in Synthesized and Sequestered Compounds in Captive-Reared Versus Wild-Caught Butterfly Species

i. Total CG concentration
  Total CG concentration in our samples was normally distributed after applying a square root transformation (Shapiro–Wilk normality test, W = 0.997, p-value = 0.246). We thus tested the effect and interaction of species (five species) and origin (captive-reared versus wild-caught) on the total CG concentrations using a two way ANOVA with stats 3.4.2 package (R Core Team, 2017). Tukey Honest Significant Differences (THSD) posthoc tests following significant ANOVA were performed.
ii. Profile of CG concentrations
  Data were not normally distributed (Shapiro–Wilk normality test, W = 0.0447, p-value  0.001) even when corrective transformation was applied. Therefore to assess the effect and interaction of species and origin (captive-reared versus wild-caught) on the profile of CG concentration, we used a two way and non-parametric PERmutational Multivariate Analysis of Variance (PERMANOVA) using the vegan 2.5–2 package (Oksanen et al., 2019).

### Experiment 2: Species, Sex, and Age-Related Differences in Chemical Defences when Controlling for Larval Host Plant Use

i. Total CG concentration
  Total CG concentration of the three butterfly species raised on the same host plant as larvae were normally distributed after applying a square root transformation (Shapiro–Wilk normality test, W = 0.979, p-value = 0.328). We tested the effect of species, sex, age, and the interaction of these factors on the total CG concentrations using ANOVA with stats 3.4.2 package (R Core Team 2017). Tukey Honest Significant Differences (THSD) posthoc tests following significant ANOVA were performed. A fitted linear model was used to visualise the correlation between age and total CG concentration for each species using stats 3.4.2 package.
ii. Profile of CG concentrations
  Data were not normally distributed (Shapiro–Wilk normality test, W = 0.707, *p-value* < 0.001), even when corrective transformation was applied. We tested the effect of species, sex, age, and the interaction of these factors on the profile of CG concentrations using non-parametric PERmutational Multivariate Analysis of Variance (PERMANOVA) using the *vegan 2.5–2* package (Oksanen et al. 2019).

### Experiment 3: Intraspecific Variation in Chemical Defences in Heliconius numata When Fed Two Different Diets.

i. Total CG concentration
  Data were normally distributed after applying a square root transformation (Shapiro–Wilk normality test, W = 0.978, p-value = 0.142). We tested the effect of larval host plant diet (n = 2), sex, age, brood line (n = 4), and the interaction of these factors on the total CG concentrations using ANOVA as detailed above.
ii. Profile of CG concentrations
  Data were not normally distributed, (Shapiro–Wilk normality test, W = 0.612, *p-value* < 0.001), even when corrective transformation was applied. We tested the effect of larval host plant diet, sex, age, brood line, and the interaction of these factors on the profile of CG concentrations using non-parametric PERMANOVA. Fitted quadratic regression models were used to visualise the correlation between the different CG compounds and age using *stats 3.4.2* package.

## Results

### Experiment 1: Differences in Synthesized and Sequestered Compounds in Captive-Reared Versus Wild-Caught Butterfly Species Showed a More Limited Range of Sequestered Compounds in Captive-Reared Butterflies

We found that the average total concentration of CGs differed between captive-reared and wild-caught butterflies (Table 2, ANOVA, F^9^_186_ = 9.930, *p* < 0.001) and that this difference is driven by *D. juno* (THSD, P = 0.003), with captive-reared individuals having 2.5 times more CGs than wild-caught butterflies of this species. Overall, in all species, the chemical diversity observed in captive-reared individuals appeared to be lower than that of wild-caught butterflies, which were found to have greater amounts and more diverse sequestered CGs (PERMANOVA, F^9^_186_ = 23.42, *p* < 0.001). Notably, sequestered CGs such as tetraphyllin A and B and dihydrogynocardin were either absent or in too low concentration to be detected in captive-reared individuals.

**Table 2:** Cg concentration (µg/mg of dry weight; mean ± se) for captive-reared adults of five heliconiini species compared with wild caught butterflies

CGs Species	Putatively-synthesized			Sequestered				Total [CGs] µg/mg of DW
	Linamarin	Lotaustralin	Epilotaustralin	Epivolkenin	Tetraphyllin B	Tetraphyllin A	Dihydrogynocardin	
*Dione juno* Reared Wild	31.77 ± 6.00 12.50 ± 2.11	7.83 ± 1.97 2.76 ± 1.08	1.90 ± 0.40 1.13 ± 0.16	0.00 0.00	0.00 0.00	0.00 0.00	0.00 0.00	41.51 ± 7.96 16.40 ± 2.72
*Heliconius doris* Reared Wild	41.15 ± 3.16 24.37 ± 2.93	2.44 ± 0.34 5.50 ± 2.60	0.02 ± 0.02 0.09 ± 0.09	0.00 0.00	0.00 0.00	0.00 0.00	0.00 0.00	43.60 ± 3.35 29.97 ± 4.77
*Heliconius erato* Reared Wild	2.82 ± 7.62 0.52 ± 0.25	1.14 ± 2.32 0.18 ± 0.12	0.37 ± 0.60 0.00	1.87 ± 0.42 6.74 ± 1.73	0.00 1.76 ± 0.68	0.00 0.07 ± 0.05	0.00 0.04 ± 0.03	6.20 ± 9.84 9.31 ± 1.19
*Heliconius melpomene* Reared Wild	21.00 ± 5.57 15.50 ± 1.68	3.16 ± 1.54 5.82 ± 0.69	1.52 ± 1.29 0.72 ± 0.18	0.00 0.46 ± 0.40	0.00 3.84 ± 0.96	0.00 0.15 ± 0.10	0.00 0.00	25.69 ± 7.61 26.48 ± 2.31
*Heliconius numata* Reared Wild	26.88 ± 2.95 14.44 ± 1.45	2.69 ± 0.38 5.29 ± 0.65	0.33 ± 0.10 0.76 ± 0.19	0.00 3.04 ± 0.11	0.00 0.13 ± 1.25	0.00 0.00	0.00 0.00	29.89 ± 3.24 23.68 ± 2.64

Total number of captive-reared adults *n* 80: *D. juno n*  20, *H. doris n* 22 and *H. numata n* 24 were reared as larvae on *Passiflora riparia, H. erato n* 9 was reared on *P. trifasciata* and *H. melpomene n* 5 was reared as larvae on *P. triloba*

Total number of wild-caught adults *n* 116: *D. juno n* 13, *H. doris n* 8, *H. erato n* 36, *H. melpomene n* 25, *H. numata n* 34

### Experiment 2: Species, Sex and Age-Related Differences in Chemical Defences when Controlling for Larval Host Plant Use: Sex Related Differences in Chemical Defences

Captive-reared adults of *D. juno, H. doris,* and *H. numata* only had putatively-synthesized CGs (Table 2), consistent with feeding as larvae on a plant (*P. riparia*) with no CGs available for sequestration. We found a difference between sexes in both the CG profile and the total concentration (Table 3), with adult females generally being better defended than adult males (*n* = 35 females, mean total [CGs] = 47.68 ± 4.44 µg/mg DW; *n* = 31 males, mean total [CGs] = 27.03 ± 2.79 µg/mg DW). This was mostly driven by the striking sexual dimorphism observed in *D. juno*, where females were on average seven times more defended than males (ANOVA, F^1^_18 =_ 94.85, *p* < 0.001; Table1, Table 2). These large chemical differences in *D. juno* may be responsible for the significant interaction found between species and sex for both the CG profile and the total concentration (Table 3). Age had no effect on CG variation among our samples (Table 3), but as the sample size is not well distributed across ages (because of larval mortality), statistical power is low.

**Table 3:** Analysis of total cg concentration (anova) and cg profile (permanova) of captive-reared individuals as a function of heliconiini species, sex and age

Tested factors	Statistical results	
Heliconiini	Total [CGs]	Profile of [CGs]
Species	F^2^_54_ = 4.469, P = 0.016*	F^2^_54_ = 6.374, P = 0.002*
Sex	F^1^_54_ = 20.080, P < 0.001*	F^1^_54_ = 18.364, P < 0.001*
Age	F^1^_54_ = 2.463, P = 0.122	F^1^_54_ = 0.822, P = 0.366
Species: Sex	F^2^_54_ = 28.183, P < 0.001*	F^2^_54_ = 18.872, P < 0.001*
Species: Age	F^2^_54_ = 1.972, P = 0.149	F^2^_54_ = 1.439, P = 0.245
Species: Sex: Age	F^2^_54_ = 0.472, P = 0.626	F^2^_54_ = 0.482, P = 0.644

Total individual numbers: *n* 66; *D. juno n* 20, *H. doris n* 22 and *H. numata n* 24. * indicates significant differences (P < 0.001)

### Experiment 3: Intraspecific Variation in Chemical Defences in *Heliconius numata* When Fed Two Different Diets: The Role of Brood Line and Larval Host Plants on Intraspecific Variation of CG in *H. numata*

**Fig. 2:**
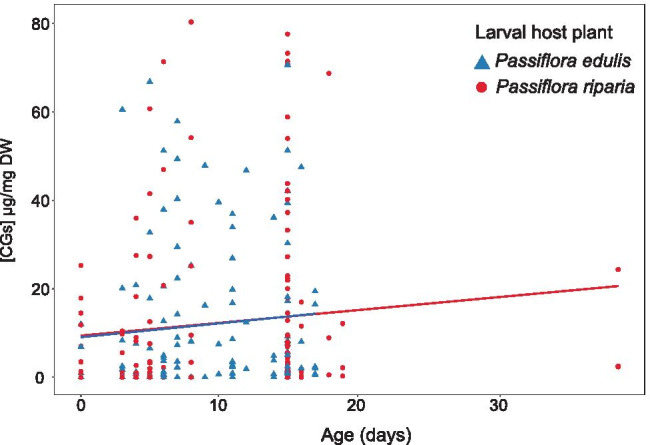
The concentration of total CG compounds (µg/mg dry weight) with increasing age in captive-reared adults of *H. numata*, as a function of the larval host plant

**Fig. 3:**
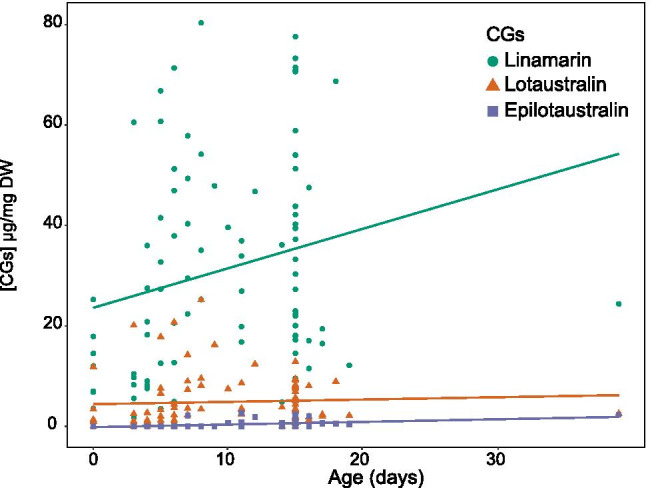
Concentration of each CG compound (µg/mg dry weight) as a function of increasing age in captive-reared adults of *H. numata*

To assess the role of larval host plant and brood lines on chemical defences, we compared the CG profiles of butterflies from four lineages of *H. numata* (Table 1) reared on two different *Passiflora* species: *P. edulis* and *P. riparia*. As mentioned previously, CG content of *H. numata* fed as larvae with *P. riparia* resulted from de novo synthesis. This is expected as this *Passiflora* species does not appear to contain any CG compounds (de Castro et al. 2019). Surprisingly, however, individuals reared on *P. edulis* as larvae did not appear to sequester tetraphyllin B, previously reported for this larval host plant (Patel 2009), except for a single female. As a result, we did not find any significant differences in chemical compounds between individuals as a function of larval diet (Table 4, Fig. 2). However, we did find a significant increase of total CG concentration with age in *H. numata* (ANOVA, F^1^_57_ = 7.536 P = 0.008; Table 4), as well as a positive correlation of compound concentration with age (PERMANOVA, F^1^_57_ = 5.317 P = 0.022; Table 4). This increase in chemical defences over time was found to be associated with an increase in the synthesized linamarin (Fig. 3, linear model: linamarin R^2^ = 0.057, F^1^_86_ = 5.193, P = 0.025). Individuals from different brood lines had statistically different total CG concentration (ANOVA, F^1^_57_ = 2.900 P = 0.043), consistent with the trait being inheritable to some extent.

**Table 4:** Analysis of total cg concentrations (anova) and cg profiles (permanova) in captive-reared adults of *h. numata* as a function of brood line, sex, larval host plants (*p. edulis* and *p. riparia*), and age

Tested factors	Statistical results	
*Heliconius numata*	Total [CGs]	Profile of [CGs]
Larval host plant	F^1^_57_ = 0.045 P = 0.832	F^1^_57_ = 0.287 P = 0.607
Sex	F^1^_57_ = 0.256 P = 0.615	F^1^_57_ = 0.611 P = 0.441
Age	F^1^_57_ = 7.536 P = 0.008*	F^1^_57_ = 5.317 P = 0.022*
Brood line	F^3^_57_ = 2.900 P = 0.043*	F^3^_57_ = 2.232 P = 0.091
Larval host plant: Age	F^1^_57_ = 0.101 P = 0.752	F^1^_57_ = 0.005 P = 0.986
Sex: Age	F^1^_57_ = 1.436 P = 0.236	F^1^_57_ = 1.436 P = 0.231
Sex: Brood line	F^3^_57_ = 1.457 P = 0.236	F^3^_57_ = 1.600 P = 0.201
Larval host plant: Brood line	F^3^_57_ = 0.449 P = 0.719	F^3^_57_ = 0.682 P = 0.575
Larval host plant: Sex: Age	F^1^_57_ = 0.470 P = 0.496	F^1^_57_ = 0.393 P = 0.545

A total of 88 captive-reared adults (44 females and 44 males) were used. * indicates significant differences (P < 0.001)

*H. numata* individuals (total *n* = 88) were fed as larvae on *P. riparia* (*n* = 41 individuals) or *P. edulis* (*n* = 47 individuals)*.* Captive-reared adult butterflies were sampled from day 0 (which is the day of emergence) to 39. Blue and red lines are regression lines on CGs for individuals reared on either *P. edulis* (y = 0.287x + 2.521, R^2^ = 0.01, F^1^_139_ = 1.635, P = 0.203) or *P. riparia* (y = 0.312x + 1.588, R^2^ = 0.01, F^1^_121_ = 0.937, P = 0.335) respectively.

*H. numata* (*n* = 88 individuals) were fed as larvae on *P. riparia* (*n* = 41 individuals) or *P. edulis* (*n* = 47 individuals)*.* Captive-reared adult butterflies were sampled from day 0 (which is the day of emergence) to 39. Regression line base on quadratic regression for linamarin (green): y = 5.251x—74.329, R^2^ = 0.116, F^2^_85_ = 5.558, P = 0.005; lotaustralin (orange): y = 0.725x—8.724, R^2^ = 0.026, F^2^_85_ = 1.132, P = 0.327; epilotaustralin (purple): y = 0.005x – 589, R^2^ = 0.266, F^2^_85_ = 15.41, *p* < 0.001.

## Discussion

Rearing butterflies in controlled conditions is essential for deciphering the role of ecological and developmental factors affecting the variation in chemical defences observed in natural populations. Indeed, extensive variation in the chemical defences of Heliconiini have been observed in the field, such that different species, but also different geographic populations of species, differ in the amount and types of compounds (Sculfort et al. 2020). For wild-caught butterflies, age and *Passiflora* larval host plant cannot easily be ascertained, and it is therefore not possible to quantify the effect of these factors on the CG variation observed. However, by comparing CG content variation of individuals sampled from natural populations and that of individuals reared in captivity, where we control for both age and larval host plant, we can estimate how much of the variation can be explained by these factors in captive-reared individuals.

### Greater Investment into Synthesized CG Compounds in Captive-Reared Butterflies Than in Wild-Caught Butterflies, and a Potentially Important Role for the Larval Host Plants.

We found substantial differences in the chemical defences of different Heliconiini species, as previously reported in other studies (Arias et al. 2016; de Castro et al. 2019; Engler and Gilbert 2007; Nahrstedt and Davis 1983, 1985; Sculfort et al. 2020), even when the different species were reared on the same host plant as larvae (see Table 3: *D. juno*, *H. doris* and *H. numata*). Interestingly, captive-reared individuals had greater amounts of CGs, with variation among species, but wild-caught butterflies had more diverse sequestered CGs (Table 2). As individuals reared in captivity had access to unlimited food and resources, they may be able to invest more resources into defences, explaining why the individuals reared in captivity had overall higher amounts of CGs. By contrast, wild individuals must face decreasing foliage quality, competition for access to food, parasites, and variable climatic conditions, all of which may limit resources and/or ability to synthesize these compounds. Interestingly, however, very few butterflies reared in captivity had sequestered compounds. This may be because in the wild, Heliconiini females of a given species have access to a large number of different host plants to lay eggs. Thus, at a species level, wild-caught butterflies are expected to have more diverse sequestered CGs as compared to captive-reared butterflies. Indeed, CG composition might strongly differ between *Passiflora* species, and the intraspecific diversity of sequestered CGs is thus expected to be higher in wild-caught butterflies than in captive-reared butterflies, fed on a single *Passiflora* species. The chemical composition of larval host plants may also depend on spatially variable ecological factors (soil quality, vegetation cover, herbivory pressure, drought, etc.), as well as temporal variation linked to seasonality (wet/rain and dry/drought seasons) (Hay-Roe and Nation 2007; but see Wheeler and Bennington 2001). Greater host plant availability and oviposition choices, in addition to the variation of chemical composition within plant species, may therefore greatly influence the defences of Heliconiini butterflies. For example, in *H. erato,* the amount of chemical defences differed as a result of the *Passiflora* species used as larval host plants, although this was only significant in females (Hay-Roe and Nation 2007). However, we did not find a significant effect of larval host plants on the chemical defences of *H. numata* butterflies reared on different *Passiflora* species. The CG biosynthesis pathway might differ between these two species, which belong to different clades of the *Heliconius* genus. The limited effect of larval host plants in *H. numata* might also be influenced by its generalist host plant use by larvae, which could be associated with greater investment in CG neo-synthesis, rather than specialized sequestration from the host plant. Further studies are certainly warranted, as chemical profiles may depend on the *Heliconius* species, diet breadth, or, as mentioned previously, qualitative differences of the plants as a result of the environmental growing conditions.

### Physiological Factors Responsible for the Variation in Cyanogenic Glucoside Profiles in Heliconiini.

 Part of the variation in chemical profiles found within species observed in our captive-reared butterflies was correlated with sex. Differences between the sexes were found to be significantly affected by species, suggesting substantial variation in CG biosynthesis pathways that may be differently influenced by sex, especially among different Heliconiini clades. This may explain why some previous studies on Heliconiini did not report sexual differences in CG concentration (Arias et al. 2016; de Castro et al. 2019) while others do (e.g. in *H. erato*, Hay-Roe 2004). Indeed, our study is the first to reveal such a large difference between females and males of *D. juno*. In *H. numata*, differences in defences were also found to be linked with age, consistent with previous studies in other species such as the closely related species *H. melpomene*, where increasing amounts of synthesized CGs with age have been observed (de Castro et al. 2020). These results thus confirm that continuous investment in CG synthesis during the entire life cycle could be widespread in Heliconiini butterflies. The ability to synthesize CG throughout life, and not only sequester them from larval host plants, may have evolved in response to predation pressure and long-life expectancies. Thus, plasticity in CG variation can stem from proximal factors (environmental and physiological), as well as evolutionary factors. Increased reliance on synthesized CGs as adults could potentially ensure sufficient continued defences, regardless of the *Passiflora* host plant fed upon during larval development. Our results suggest that de novo synthesis in adults may be more important to overall adult chemical defences than previously believed, and that differences in synthesis and/or sequestration abilities may be responsible for intraspecific variation and therefore naturally occurring differences within wild populations.

### Inheritability (i.e. Genetic and/or Maternal Effect) of Chemical Defences.

 Our experiment specifically testing differences in lineages of *H. numata* shows some degree of inheritability of the CG content of butterflies. These putatively inherited differences could be the result of genetic differences, as seen in other Lepidoptera species relying on CGs for defences (Witthohn and Naumann 1987; Wray et al. 1983; Zagrobelny et al. 2007a, b). Alternatively, this could also stem from differences in the amount of resources transmitted by the mother. Indeed, the amino acids in the eggs are provided by the female (O’Brien et al. 2003) and these compounds are CG precursors (Cavin and Bradley 1988; Nahrstedt and Davis 1983). Thus, maternal transmission may also explain some of the observed chemical variation between individuals and species.

In conclusion, we investigated the qualitative and quantitative differences in defensive compounds found among and within different Heliconiini species and attempted to elucidate the causes for the variability observed in wild populations. We found a significant effect of species, driven by *Dione juno,* on CG content between captive-reared and wild-caught species. Also, in captive-reared butterflies, CG content varies significantly between species, even when they were reared on the same larval host plant. Overall, we found individuals reared in captivity had higher concentrations of CGs than wild individuals did, but had fewer sequestered compounds. This decreased diversity of compounds is likely the result of qualitative and quantitative differences in larval host plants, and although no effect of larval host plant was found in the generalist *H. numata*, further studies are warranted. Unlike previously suggested, the effect of sex differed between species and should therefore be taken into consideration in future studies. In some species, chemical defences continue to increase all through life, and this appears to be the result of continued biosynthesis. Finally, our comparison of different brood lines in *H. numata* suggests that the amount of synthesized CGs is an inheritable trait. Our results should therefore stimulate further studies on the evolution of chemical defences, and the underlying physiological, ecological and inheritable factors that affect them.

## Data Availability

Raw data file describing each compound and concentration per individual will be uploaded on Dryad once the manuscript is accepted.
